# Photodynamic Vaccination of BALB/c Mice for Prophylaxis of Cutaneous Leishmaniasis Caused by *Leishmania amazonensis*

**DOI:** 10.3389/fmicb.2018.00165

**Published:** 2018-02-06

**Authors:** Sayonara M. Viana, Fabiana S. Celes, Laura Ramirez, Bala Kolli, Dennis K. P. Ng, Kwang P. Chang, Camila I. de Oliveira

**Affiliations:** ^1^Instituto Gonçalo Muniz (IGM), FIOCRUZ, Salvador, Brazil; ^2^Department of Microbiology/Immunology, Chicago Medical School, Rosalind Franklin University of Medicine and Science, North Chicago, IL, United States; ^3^Department of Chemistry, The Chinese University of Hong Kong, Hong Kong, Hong Kong; ^4^Instituto Nacional de Ciência e Tecnologia (iii-INCT) - Instituto de Investigação em Imunologia, São Paulo, Brazil

**Keywords:** *Leishmania*, leishmaniasis, potosensitizer, phthalocyanine, uroporphyrin, photodynamic vaccination, suicidal vaccination, cutaneous leishmaniasis

## Abstract

**Background:** Photosensitizers (PS), like porphyrins and phthalocyanines (PC) are excitable by light to generate cytotoxic singlet oxygen and other reactive oxygen species in the presence of atmospheric O_2_. Photodynamic inactivation of *Leishmania* by this means renders them non-viable, but preserves their effective use as vaccines. *Leishmania* can be photo-inactivated after PS-sensitization by loading via their endocytic uptake of PC or endogenous induction of transgenic mutants with delta-aminolevulinate (ALA) to accumulate cytosolic uroporphyrin I (URO). Here, PS-sensitization and photo-inactivation of *Leishmania* *amazonensis* was further examined *in vitro* and *in vivo* for vaccination against cutaneous leishmaniasis (CL).

**Methods and Results:** *Leishmania amazonensis* promastigotes were photodynamically inactivated *in vitro* by PC-loading followed by exposure to red light (1–2 J/cm^2^) or ALA-induction of uroporphyrinogenic transfectants to accumulate cytosolic URO followed by longwave UV exposure. When applied individually, both strategies of photodynamic inactivation were found to significantly, albeit incompletely abolish the MTT reduction activities of the promastigotes, their uptake by mouse bone marrow-derived macrophages *in vitro* and their infectivity to mouse ear dermis *in vivo*. Inactivation of *Leishmania* to completion by using a combination of both strategies was thus used for the sake of safety as whole-cell vaccines for immunization of BALB/c mice. Different cutaneous sites were assessed for the efficacy of such photodynamic vaccination *in vivo*. Each site was inoculated first with *in vitro* doubly PS-sensitized promastigotes and then spot-illuminated with white light (50 J/cm^2^) for their photo-inactivation *in situ*. Only in ear dermis parasites were photo-inactivated beyond detection. Mice were thus immunized once in the ear and challenged 3 weeks later at the tail base with virulent *L. amazonensis*. Prophylaxis was noted in mice photodynamically vaccinated with doubly photo-inactivated parasites, as indicated by a significant delay in the onset of lesion development and a substantial decrease in the parasite loads.

**Conclusion**: *Leishmania* doubly PS-sensitized and *in situ* photo-inactivated as described proved to be safe and effective when used for one-time immunization of ear dermis, as indicated by its significant protection of the inherently very susceptible BALB/c mice against CL.

## Introduction

Cutaneous leishmaniasis (CL) is caused by protozoan parasites in the genus of *Leishmania* and is a wide-spread disease, with estimated 1.5 million new cases per year (WHO, 2010). CL presents a varied spectrum of clinical manifestations that are determined presumably by both the type and magnitude of the human immune responses as well as by the differences of the causative agents (Reithinger et al., 2007). *Leishmania* infection frequently produces no clinical symptom, but sometimes causes a localized lesion, characteristic of simple CL and also more severe diseases, i.e., diffused CL and mucosal leishmaniasis [reviewed in (Bittencourt et al., 1993)]. Clinical management of leishmaniasis has been based solely on treatment of patients by chemotherapy with antiquated and toxic drugs that elicits resistance (Yasinzai et al., 2013), thus making the development of an effective vaccine all the more urgent.

Immunologically competent individuals after recovery from leishmaniasis develop lifelong immunity, indicative of the feasibility to develop an effective prophylactic vaccine. It is possible to elicit protective immunity to human CL by leishmanization, i.e., inoculation of healthy individuals with a low dose of live *Leishmania* (Nadim et al., 1983). Leishmanization is, however, unacceptable because of its association with the development of non-healing lesions, especially in immunocompromised individuals [reviewed in (Palatnik-De-Sousa, 2008)]. Attempts to overcome these difficulties included the use of parasites after attenuation via, for example, long-term *in vitro* cultivation (Daneshvar et al., 2003), genetic modifications (Alexander et al., 1998; Spath et al., 2000; Uzonna et al., 2004; Selvapandiyan et al., 2009; Dey et al., 2013; Bhattacharya et al., 2015) and gamma irradiation (Alexander, 1982). Although such attenuated parasites immunologically protect susceptible animals against experimental challenges, the risk of potential reactivation remains to be a concern for their clinical use, especially among immunocompromised individuals (Sundar and Singh, 2014).

We have explored the principle of photodynamic therapy (PDT) as a new strategy for *Leishmania* inactivation *in vitro* to develop non-viable, but immunologically competent whole cell vaccines and vaccine carriers (Sah et al., 2002; Dutta et al., 2005; Chang and Kolli, 2016). PDT uses photosensitizers (PS) that are excitable by light at a specific wavelength to produce reactive oxygen species (ROS) for the clinical treatment of skin diseases, such as psoriasis (Chen et al., 2017), actinic keratosis (Canavan et al., 2017), carcinoma (Wang et al., 2016) and CL (Enk et al., 2015). Our attention to PDT started with the work on *Leishmania* genetic deficiency in the enzymes of heme biosynthesis. *Leishmania* spp., e.g., *Leishmania amazonensis* were genetically complemented to express the 2nd and 3rd enzymes in this biosynthetic pathway, i.e., delta-aminolevulinate (ALA) dehydratase (ALAD) and porphobilinogen deaminase (PBGD). Upon exposure of these mutants to ALA, uroporphyrin I (URO) accumulates in the cytosol, rendering them light sensitive as a PS to generate cytotoxic singlet oxygen (^1^O_2_) and other ROS (Sah et al., 2002; Dutta et al., 2008). This strategy of photo-inactivation, especially in combination with additional sensitization with exogenous phthalocyanines (PC) irreparably damages all *Leishmania* cells. Significantly, repeated cycles of PDT selected no PDT-resistant mutants (Dutta et al., 2011). These and other properties of PDT argue strongly in favor of its use to generate inactivated parasites for vaccination, especially for eliciting cell-mediated immunity via oxidative and proteolytic processing of vaccines in macrophages and other antigen-presenting cells (APC) for epitope presentation to the immune system. Indeed, vaccination of hamsters with porphyrinogenic *L. amazonensis* followed by *in vivo* ALA treatment and light exposure conferred protection on these susceptible animals against the challenge with virulent *L. donovani* (Kumari et al., 2009). Significantly, this immunity is adaptively transferrable from immunized hamsters to naïve animals.

In the present study, we have evaluated initially both endogenous and exogenous strategies separately for photo-inactivation of *L. amazonensis* based on parasite viability, parasite uptake *in vitro* and lesion development in mice. Only when doubly PS-sensitized with exogenously provided PC together with endogenously generated URO, were promastigotes rendered susceptible to complete photo-inactivation by spot-illumination *in situ*, but only in the ear dermis. Ear dermis was thus the site chosen for immunization of BALB/c mice. This photodynamic vaccination prophylactically protects the highly susceptible strain of mice against challenge infection, as indicted by the delay in lesion development and reduction in parasite loads.

## Materials and Methods

### Ethics Statements

Female BALB/c mice, 6–8 weeks of age, were obtained from CPqGM/FIOCRUZ animal facility where they were maintained under pathogen-free conditions. All animal work was conducted according to the Guidelines for Animal Experimentation of the Colégio Brasileiro de Experimentação Animal and of the Conselho Nacional de Controle de Experimentação Animal. The local Ethics Committee on Animal Care and Utilization (CEUA) approved all procedures involving animals (CEUA-003/2014-IGM/FIOCRUZ).

### Parasites

*Leishmania amazonensis* (MPRO/BR/72/M1845/LV78) clone 12-1 was maintained as promastigotes in Medium 199 (SIGMA) containing 10% heat-inactivated fetal bovine serum (FBS), 2 mM L-glutamine and antibiotics (penicillin 100 IU/mL and streptomycin 100 μg/mL) (all from Invitrogen). Genetically complemented *L. amazonensis* expressing ALAD and PBGD (Sah et al., 2002) were grown as described above in the presence of G418 (100 μg/mL) (Sigma) and tunicamycin (20 μg/mL) (CalBiochem). Before exposure of the transfectants to ALA for uroporphyrinogenesis, they were grown for one-cycle in drug-free medium to avoid potential cytotoxicity of the carryover drugs to macrophages during *in vitro* and *in vivo* infection.

### *In Vitro* PS-Sensitization and Photo-Inactivation of *Leishmania*

The exogenous PS used for this study included amino-phthalocyanines, e.g., PC2 (Al-Qahtani et al., 2016) and aluminum phthalocyanine chloride (AlPhCl, Sigma) (Dutta et al., 2005). Photosensitizers were dissolved in dimethyl sulfoxide (DMSO) (SIGMA) as 1 mM stock solutions. For exogenous sensitization, *L. amazonensis* promastigotes were grown to late-log phase, washed and resuspended in Hank’s Balanced Salt Solution (Invitrogen)/0.01% bovine serum albumin (HBSS-BSA), pH 7.4, in presence of the PC (0.1–10 μM). Cells exposed to diluent (DMSO) equivalent to the highest PC concentration were used as controls. After overnight incubation at 26°C in the dark, PC-sensitized and control cells were washed and illuminated with red light (RL) until the cessation of their flagellar motility (1–2 J/cm^2^) as described (Dutta et al., 2011).

For endogenous PS-sensitization, *L. amazonensis* genetically complemented to express ALAD and PBGD (Sah et al., 2002) were exposed to 1 mM ALA (SIGMA) in HBSS-BSA for 24–48 h at 26°C in the dark for accumulation of cytosolic URO (Dutta et al., 2008). URO-loaded *L. amazonensis* were washed, placed in unlidded wells and then exposed to longwave UV (λ_max_ = 365 nm) from the top for 20 min as before. Uroporphyric cells kept in the dark served as controls.

### Microscopy, MTT Reduction and Growth Assays

The effect of exogenous (PC) and endogenous (ALA-URO) PS-sensitization with and without photo-inactivation on *L. amazonensis* was examined by phase contrast and fluorescence microscopy for PC and porphyrin using filter sets previously described (Dutta et al., 2008). After incubation for PS-loading (overnight for PC and 24–48 h for ALA-URO), one set of samples were kept in the dark and the other set exposed to light at the excitation wavelengths specific to PC or URO, also as described before (Sah et al., 2002; Dutta et al., 2011). All cell samples were subjected to MTT [3-(4,5-dimethylthiazol-2-yl)-2,5-diphenyltetrazolium bromide] reduction assay (SIGMA) according to the manufacturer’s protocol. Treated and control cells (2 × 10^5^) were also inoculated into Schneider medium containing 20% FBS for growth, as determined by daily enumeration of cell density in a haemacytometer in quintuplicate.

### *In Vitro* Uptake of PS-Sensitized and Photo-Inactivated *L. amazonensis* by Bone Marrow-Derived Macrophages (BMDM)

The macrophages were obtained as previously described (Weischenfeldt and Porse, 2008), resuspended in RPMI 1640 medium (SIGMA) supplemented with 100 U/ml penicillin, 100 μg/ml streptomycin and 10% FBS for seeding onto glass coverslips at 3 × 10^5^ cells/coverslip placed in 24-well plates. Monolayers formed on the coverslips were each infected with 3 × 10^6^ control or experimental cell samples (10:1 parasite/host cell) in RPMI 1640 containing 20% FBS at 35°C, 5% CO_2_. After 4 h, monolayers were extensively washed to remove non-internalized parasites, fixed and stained with hematoxylin and eosin. Parasite uptake was determined by microscopic counting of 200 macrophages in quintuplicate for the number of infected cells, non-infected cells and intracellular *Leishmania*.

### Inoculation of BALB/c Mouse Ear Dermis with *in Vitro* Singly PS-Sensitized and Photo-Inactivated *L. amazonensis*

Photosensitizers-sensitized promastigotes of *L. amazonensis* with and without photo-inactivation *in vitro* were inoculated into the ear dermis of BALB/c mice, each with ∼10^6^ cells using a 27.5-gauge needle. Ear thickness was measured periodically by using a digital caliper (Thomas Scientific).

### Inoculation of BALB/c Mice at Different Cutaneous Sites with *in Vitro* Doubly PS-Sensitized *Leishmania* for *in Situ* Photo-Inactivation to Select Suitable Site for Immunization

Promastigotes of the mutant *L. amazonensis*, which were doubly sensitized in the dark with PC (AlPhCl, 0.1 μg/ml) and ALA/URO were inoculated into four groups of BALB/c mice at different cutaneous sites: ear dermis, shaved flank or back, footpad and tail base. Each group consisted of four mice, each inoculated subcutaneously in the given location with 10^6^ parasites. The use of this cell number was chosen as the most adequate size of inoculation based on prior testing of 10^3^ to 10^7^ per site. After 24 h, each site received an additional injection of 100 mM ALA (100 μl) to boost uroporphyrinogenesis of the inoculated transfectants *in situ*. After another 36 h, a set of mice was spot-illuminated (individually at the inoculation site) with white light generated from a probe, consisting of heatless fiber optic end-point emitter at 50 J/cm^2^ (LumaCare model LC122, MGB Technologies, Inc.); the other set of mice received no spot-illumination. All mice were inspected every other day at the inoculated sites for lesion development. After 3 weeks, mice were euthanized and tissues surrounding the injection sites were removed and homogenized. The homogenates were subjected to limiting dilution assay in 96 wells for growth to estimate the number of surviving parasites (Dutta et al., 2012).

### Ear Dermis Immunization of BALB/c Mice with *in Vitro* Doubly PS-Sensitized *L. amazonensis* for Their *in Situ* Photo-Inactivation Followed by Challenge Infection

The choice of immunization site and dosage was based on the outcome of the experiments described in the preceding section (see section “Results”). Porphyrinogenic transfectants of *L. amazonensis* were doubly PS-sensitized *in vitro* with ALA (1 mM) and AlPhCl (0.1 μM). The PS-sensitized cells were washed and resuspended to 10^9^ cells/ml in HBSS-BSA. Controls were similarly prepared, consisting of six different groups: untreated cells exposed to light (*La*+Light), singly PS-sensitized cells without light (*La*+ALA; *La*+AlPhCl), singly PS-sensitized cells with light (*La*+ALA+Light; *La*+AlPhCl+Light) and both PS alone with light (ALA+AlPhCl+Light). There were thus seven groups, each consisting of six BALB/c mice. Each mouse was immunized once in the ear dermis with the experimental or one of the six control cell samples at 10^6^ parasites/10 μl HBSS-BSA/mouse. One day later, an additional volume (∼100 μl in total) of 100 mM ALA was injected into the ear dermis. After 36 h, ear dermis was exposed to white light (50 J/cm^2^) in five of the seven groups according to the experimental designs indicated. Experimental and control mice were each challenged 3 weeks later with 10^7^ stationary-phase *L. amazonensis* promastigotes at the tail base. Lesion size in diameter was measured periodically for a total period of ∼10 weeks post challenge. Parasite loads at the challenge sites were determined by limiting dilution assay of the parasites in the tissues at the end point of the experiment.

### Data Analysis

Comparisons between two groups were performed by Mann–Whitney (non-parametric *t*-test) and comparisons among more than two groups were performed by Kruskal–Wallis. Analyses were conducted using Prism (GraphPad, V 5.0) and a *p-*value ≤ 0.05 was considered significant. The course of disease for mice in all experimental and control groups was plotted individually. Disease burden was calculated in some cases as the diseased area under the curve (AUC). Lesion development was assessed by measuring thickness or lesion diameter, depending on the site of inoculation in ear dermis or tail base. Data are presented as mean ± standard deviation.

## Results

### PC-Sensitized and Photo-Inactivated *L. amazonensis* Lost Their Viability Substantially, but Remain Susceptible to Endocytosis by Mouse Bone Marrow Macrophages *in Vitro*

**FIGURE 1:**
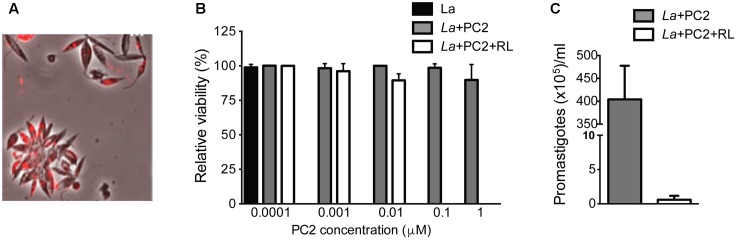
PC2-mediated photo-inactivation of *Leishmania amazonensis*. Promastigotes (La) were incubated in the dark with PC2 for 16 h and exposed to red-light (RL) (=1–2 J/cm^2^). **(A)** Merged phase contrast and Cy5 fluorescence images, showing the uptake of PC2 by cells sensitized in the dark overnight with 1 μM PC2. **(B)** MTT reduction activities of cells sensitized with graded concentrations of PC2, as indicated, in the dark (gray bar) and after light exposure (white bar), the values being expressed in % of untreated controls (black bar). **(C)** Disparity in cell density between PC2-sensitized cells with (white bar) and without photo-inactivation (gray bar) after inoculation into culture medium. Similar results were obtained after PS-sensitization with either 0.1 or 1 μM PC2. Data are presented as mean ± SD from a representative set of experiments performed in quadruplicate.

*Leishmania amazonensis* were PC-sensitized and exposed to red light for photo-inactivation, e.g., amino-PC2. Fluorescence microscopy of live promastigotes showed PC2 localization in cytoplasmic vacuoles (**Figure 1A**). Without light exposure, these PC-loaded cells remained, as expected, intact and motile, just like the untreated controls. By MTT reduction assays, PC-loaded (e.g., 0.1–10 μM PC2) cells were shown to lose their viability only after light exposure in contrast to parasites kept in the dark (**Figure 1B**, white bars vs. gray bars). When sensitized with 0.1 μM PC and exposed to light, cells lost flagellar motility; except very few, which failed to grow up on further incubation under the experimental conditions described (*p* = 0.0286; **Figure 1C**). The ED_50_ of the amino-PC for photo-inactivation of these cells falls in between 10–100 nM according to the results with PC2 from both cell viability assays.

**FIGURE 2:**
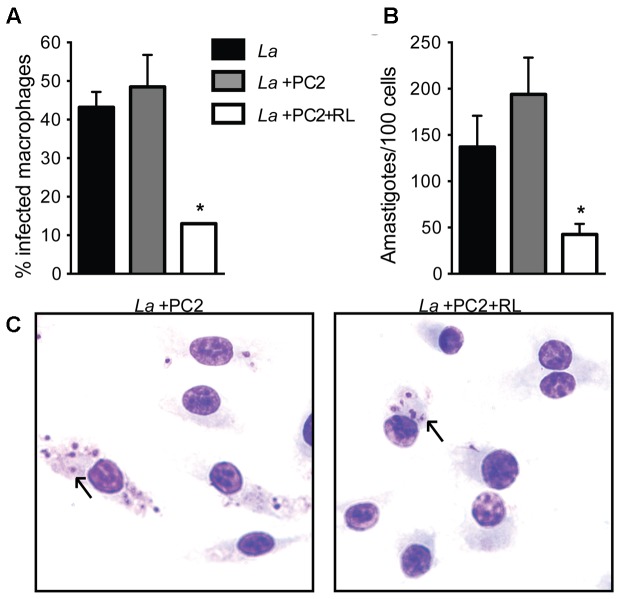
Reduced uptake of PC2-sensitized and photo-inactivated *L. amazonensis* by primary macrophages. Bone marrow-derived macrophages were infected with *L. amazonensis* (La) (black bar) or PC2-sensitized parasites with (white bar) and without (gray bar) exposure to red light (RL) at a host-parasite ratio of 1:10 for 4 h. Cells were processed for microscopy to assess: **(A)** the percentage of infected macrophages; and **(B)** the number of *Leishmania* per 100 macrophages. **(C)** Representative photomicrographs of **(A,B)**. Data are presented as mean ± SD from a representative experiment performed in quadruplicate (Kruskal–Wallis test, ^∗^*p* < 0.05). Arrow, endocytosed *Leishmania*.

Under the experimental conditions used, PC-loaded and photo-inactivated *L. amazonensis* promastigotes were taken up by BMDM but the uptake was reduced by three–fourfold in comparison to the untreated or PC-loaded controls without light exposure, judging from the rates of uptake (15% vs. 45%) (**Figure 2A**, white vs. gray and black bars) and parasite number/100 cells (50 vs. 150–200, *p* < 0.05) (**Figure 2B**, white vs. gray and black bars). Light microscopy of these samples confirms the observations as described (**Figure 2C**, *La*+PC2+RL vs. *La*+PC2). Thus, under the conditions described, PC-mediated photo-inactivation of *L. amazonensis* reduces viability of the cell population to the extent of no apparent growth when cultured *in vitro* and diminished their uptake by primary macrophages within the time frame of the experiments.

### PC-Sensitization and Photo-Inactivation of *L. amazonensis* *in Vitro* Significantly Reduces, But Does Not Eliminate Its *in Vivo* Disease-Causing Capacity

**FIGURE 3:**
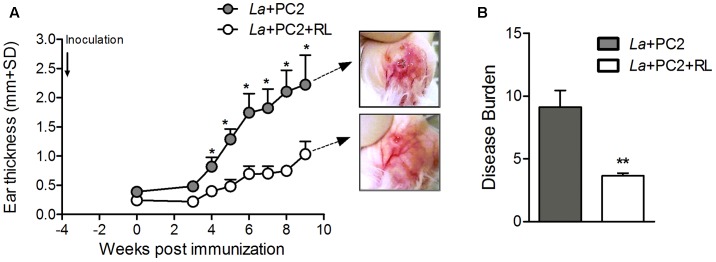
Lesion development in mice inoculated with *in vitro* PC2-sensitized/photo-inactivated *L. amazonensis*. BALB/c mice were inoculated with PC2-sensitized *L. amazonensis* (La) with (open circle/white bar) and without (gray circle/gray bar) prior red light exposure (RL). **(A)** Ear thickness was measured weekly for 9 weeks. Photomicrographs show differential severity in the appearance of the ear lesions at the end point. **(B)** Disease burden calculated by mapping the diseased areas under the curves (AUC) in **(A)**. Data are presented as mean ± SD from control and experimental groups, each consisting of six mice (^∗^*p* < 0.05, ^∗∗^*p* < 0.001, Mann–Whitney test).

Lesion development was assessed after inoculating the ear dermis of BALB/c mice with *in vitro* PC-sensitized and photo-inactivated *L. amazonensis* vs. controls not submitted to photoinactivation under otherwise identical experimental conditions. Periodic measurements of the lesions for 9 weeks showed that those produced by the controls developed much more rapidly than those by the photo-inactivated parasites, reaching >2 mm and ∼1 mm in ear thickness at the end point, respectively. This is clearly indicated by mapping the disease burden (AUC) (shown in **Figure 3A**) (*p* < 0.01), confirming that CL produced by the PC-sensitized and photo-inactivated parasites was at least twofold less severe than that produced by the controls (**Figure 3B**, clear vs. gray bar). Therefore, PC-mediated photo-inactivation of *L. amazonensis* diminishes the parasite ability to cause disease *in vivo*.

### ALA-Uroporphyric and Photo-Inactivated *L. amazonensis* Lost Their Viability, but Remain Susceptible to Endocytosis by Mouse Bone Marrow Macrophages *in Vitro*

Genetically complemented *L. amazonensis* exposed to ALA accumulated cytosolic URO, as shown by fluorescence microscopy of live promastigotes (Supplementary Figure 1A). Without UV exposure, ALA-induced uroporphyric cells remained intact and motile, similar to untreated controls, corroborating earlier findings (Sah et al., 2002; Dutta et al., 2008). By MTT reduction assays, the uroporphyric cells were shown to lose their viability after UV exposure in contrast to cells kept in the dark (Supplementary Figure 1B, clear vs. gray bar). Uroporphyric *L. amazonensis* exposed to UV light also became immobilized and reduced to such a small number that few survivors failed to grow within the time frame under the experimental conditions described (Supplementary Figure 1C, *p* = 0.002).

Under the experimental conditions established, photo-inactivated uroporphyric *L. amazonensis* promastigotes were taken up by BMDM macrophages but the uptake was reduced in comparison to no-light controls, judging from rates of uptake (<10% vs. ∼30%) (Supplementary Figure 2A) and from the parasite number per 100 macrophages (∼10 vs. >40, *p* > 0.05) (Supplementary Figure 2B, white vs. gray bar and black bar). Light microscopic examinations of these samples confirm the observations as described (Supplementary Figure 2C, *La*+ALA+UV vs. *La*+ALA). These inactivated parasites produced similar outcome as those after PC-mediated photo-inactivation when inoculated into the mouse ear dermis under the same conditions (data not shown).

### Determination of Ear Dermis as the Best Site for Photodynamic Vaccination (PDV) with *in Vitro* Doubly PS-Sensitized *Leishmani*a Followed by *in Vivo* Photo-Inactivation

**FIGURE 4:**
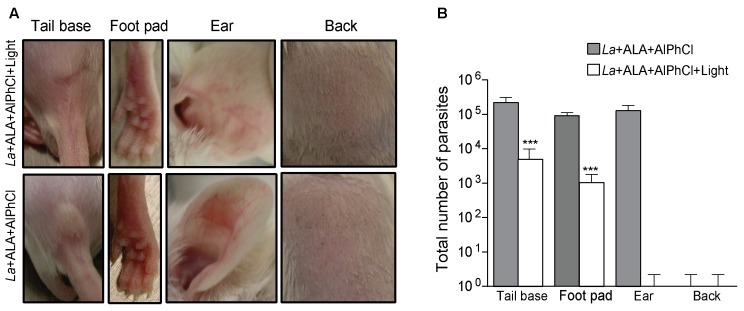
*In situ* photo-inactivation of *in vitro* doubly PS-sensitized *L. amazonensis* abolishes lesion development and parasite loads in the ear dermis of BALB/c mice. Transgenic *L. amazonensis* promastigotes (La) doubly PS-sensitized *in vitro* with URO via ALA (1 mM) treatment and AlPhCl (0.1 μM) were inoculated at 10^6^ parasites per site into different cutaneous sites as indicated. After 24 h, each site received an additional ∼100 μL of ALA (100 mM) to boost uroporphyrinogenesis. After another 36 h, the sites were spot-illuminated with white light (50 J/cm^2^). **(A)** Representative photographs of ear, tail base, footpad and back of mice 3 weeks after inoculation with experimental samples (*La*+AlPhCl+ALA+Light) (upper panel) and “no-light” controls (*La*+AlPhCl+ALA) (lower panel). **(B)** Limiting dilution of homogenates from tissues of the inoculation sites to estimate the parasite loads at the end points for experimental samples (white bar) and no-light controls (dark bar). Data are presented as mean ± SD for all sites from both control and experimental groups, each consisting of four mice per group. ^∗∗∗^*p* < 0.001.

When mice were inoculated with 10^6^ of *in vitro* doubly PS-sensitized, but not photo-inactivated *L. amazonensis* (“no light” control groups), lesions developed within the time frame of 3 weeks at all cutaneous sites, except the back (**Figure 4A** lower panel: tail base, foot pad, ear). Back was eliminated from further consideration as a site for photodynamic vaccination, since the absence of lesion in the “no-light” control group raised the uncertainly of whether the inoculated PS-sensitized parasites remained in sufficient number or remained as viable target in this site for subsequent photo-inactivation by *in situ* illumination. In the remaining three inoculation sites (tail base, footpad, and ear), lesions were produced in all the “no-light” control groups, indicative of the retention of the PS-sensitized parasites in these sites. *In situ* spot-illumination of these sites shortly after inoculation to target the PS-sensitized parasites therein for photo-inactivation produced different outcome: lesions still developed, albeit less severe in the tail base and footpad, but not at all in the ear dermis (**Figure 4A** upper panel: tail base, footpad, and ear). Lesion development or the lack of it provided a valid criterion for the efficacy of *in situ* photo-inactivation of the parasites therein, as indicated by quantitative analysis of parasite loads in inoculated sites with and without *in situ* photo-inactivation. In all three inoculation sites (tail base, footpad, and ear) examined at the end point, the parasite load per site was estimated as∼10^5^ without *in situ* photo-inactivation (**Figure 4B**, gray bar). *In vivo* photo-inactivation rendered the parasites virtually undetectable in the ear dermis, but only reduced the parasite loads by 1–2 logs in the tail base and foot pad (**Figure 4B**, white bar).

### Protection of BALB/c Mice against *L. amazonensis* by Immunization of Their Ear Dermis with *in Vitro* Doubly PS-Sensitized Parasites for *in Vivo* Photo-Inactivation

**FIGURE 5:**
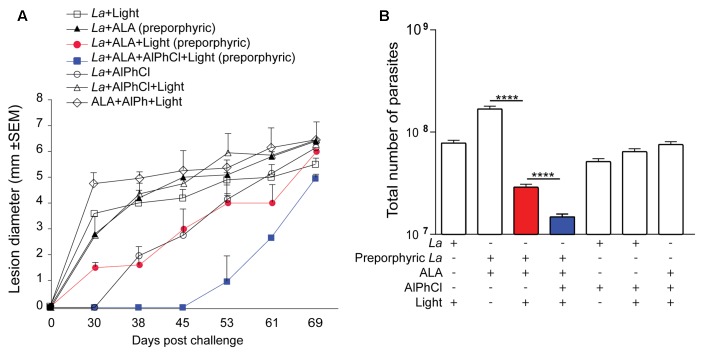
Prophylactic activities of photodynamic vaccination in ear dermis against cutaneous leishmaniasis in BALB/c mouse. Uroporphyrinogenic transfectants of *L. amazonensis* promastigotes (La) were doubly PS-sensitized *in vitro* with URO via induction by ALA (1 mM) (preporphyric) and AlPhCl (0.1 μM) for inoculation at 10^6^ cells per site in the ear of mice. After 24 h, their uroporphyrinogenesis was boosted by an additional shot of ALA (100 μL of 100 mM) in the surrounding tissue. After an additional 36 h, vaccination sites were individually illuminated *in situ* with white light (50 J/cm^2^) (Light). The experimental group of photodynamic vaccination is designated as *La*+ALA+AlPhCl+Light (Solid blue square) that is controlled by six additional groups as indicated. Mice of all seven groups were each challenged 3 weeks later at the tail base with 10^7^ stationary-phase *L. amazonensis* promastigotes. Data are presented as mean ± SD from all groups with six mice per group. **(A)** Lesion size measured periodically for a total period of ∼10 weeks post challenge. **(B)** Parasite loads estimated by limiting dilution assay for the parasites in the tissue homogenates from the challenge sites at the end point of the experiment, day 69. (*La*-ALA) vs. (*La*+ALA+Light), *p* < 0.017. (*La*+ALA+Light) vs. (*La*-ALA+AlPhCl+Light), *p* < 0.002.

Groups of BALB/c mice were photodynamically vaccinated once accordingly in the ear dermis with multiple controls and challenged 3 weeks later at the tail base under the conditions as described in Section “Materials and Methods.” Periodic measurements of lesion development with time for up to ∼10 weeks (69 days) showed that doubly PS-sensitized parasites followed by *in situ* photo-inactivation conferred the best protection. This was evident in comparison to all the control groups by a significant delay in lesion emergence by at least 14 days (**Figure 5A**, blue square) and a significant reduction of the parasite loads as the lowest of all in the challenge site at the end point (**Figure 5B**, blue bar). Single PS-sensitization of parasites with ALA-URO plus *in situ* photo-inactivation was moderately effective, as indicated by a significant reduction of the parasite loads as the second lowest of all in the challenge site at the end point (**Figure 5B**, red bar). Among the control groups, there were some variations in lesion development and slight differences in the parasite loads at the end point (**Figures 5A,B**), but no significant protection. Together, these data illustrate that immunization of BALB/c mice with doubly sensitized *L. amazonensis* for photo-inactivation by spot illumination *in situ* is safe and effective on account of no parasites recoverable from the site of vaccination and a markedly delayed onset for the emergence of the lesion with much reduced parasite loads against challenge infection.

## Discussion

Photodynamic inactivation of microorganisms such as *Leishmania* can be achieved through the intervention of PS, i.e., URO over-produced endogenously or uptake of PC provided exogenously, both being excitable by light to generate ^1^O_2_. ^1^O_2_ is highly reactive and thus extremely destructive, but too short-lived to cross the plasma membrane of cells, like *Leishmania*, allowing them to maintain structural integrity for extended time before disintegration [reviewed in (Chang and Kolli, 2016)]. Photodynamic inactivation of *Leishmania* has been studied as a new approach for producing non-viable, but immunologically competent whole-cell vaccines for immunization, akin to leishmanization to elicit effective immunity. In this study, we evaluated individually exogenous (PC supplementation) and endogenous (Uroporphyrinogenesis) photodynamic inactivation of *L. amazonensis* based on their viability, uptake by primary macrophages and disease development *in vivo*.

While excitation of all PC by red light is known to generate ^1^O_2_ (Cook et al., 1995), the amino-phthalocyanines, e.g., PC2 are 10–40 times more effective to mediate photo-inactivation of *L. tropica* (Al-Qahtani et al., 2016) than other PCs against *L. amazonensis* (Dutta et al., 2011). We extended this finding by showing that the amino-PC also dose-dependently mediates photo-inactivation of *L. amazonensis*, as seen by their loss of cell motility and viability based on microscopic observations and MTT reduction assay, respectively (**Figure 1**). PC-sensitization and photo-inactivation of *L. amazonensis* also reduced its uptake by primary macrophages (**Figure 2**). As shown here, amino-PC-sensitization of *L. amazonensis* is stochastic, leaving a small number of parasites un-sensitized, hence escaping from the fate of photo-inactivation. Stochasticity of single-PS sensitization has been reported previously, including the use of other PC (Dutta et al., 2005, 2008) and ALA induction of cytosolic URO accumulation (Sah et al., 2002; Dutta et al., 2008). This is the case despite amino-PC being much more potent than other PS to mediate *Leishmania* photo-inactivation. The development of lesion, albeit with a delayed onset and significantly reduced disease burden (**Figure 3**) is thus not unexpected after inoculation of BALB/c mice with amino-PC-sensitized and photo-inactivated *L. amazonensis*.

The potential use of single PS-sensitized and photo-inactivated *Leishmania* for PDV raises concern in considering their safety, but not efficacy. The safety is clearly a concern when lesions emerge after inoculation of these inactivated *Leishmania* into mice (**Figure 3**). The reduction of their uptake by primary macrophages *in vitro* (**Figure 2**) also may be taken to indicate diminished “vaccine” loading of the APC when applied *in vivo*. However, after such single-PS photo-inactivation, *Leishmania* transgenically modified to express OVA was shown to effectively deliver this T-cell antigen to macrophages and dendritic cells (DC) for processing and presentation to OVA-epitope specific CD8^+^ T cells *in vitro* (Dutta et al., 2011, 2012). Interestingly, immunization of Syrian Golden hamsters with single PS-sensitized and photo-inactivated *L. amazonensis* *in vivo* was shown to protect them against challenge infection with *L. donovani*: the splenic parasite load was drastically reduced by 99%, concomitant with significant increase in the expression of iNOS, IFN-γ, and IL-12 (Kumari et al., 2009). Significantly, this immunity is adaptively transferable with T cells from immunized hamsters to naïve animals, indicating that it requires no stimulation by persisting parasites, as they are unlikely to exist in the T-cell recipients. In contrast, persistence of parasites in small number cannot be ruled out in the hamsters after primary immunization with *Leishmania* subjected to single PS-sensitization/photo-inactivation, which invariably leaves behind few survivors, regardless of the PS used. Thus, while the use of incompletely photo-inactivated *Leishmania* is unacceptable for safety consideration, the results obtained point to the efficacy of PDV as a right path to vaccination.

In keeping with the synergism of two different photosensitizers in combination to enhance PDT efficacy against cancer (Schneider-Yin et al., 2009; Villanueva et al., 2010; Gyenge et al., 2013; Acedo et al., 2014), we previously showed that *L. amazonensis* doubly sensitized with URO+PC *in vitro* was fully susceptible to *in vivo* photo-inactivation in BALB/c X C57BL/6 mice, producing no lesion and no detectable parasites 8 weeks after inoculation with 10^6^ cells per site into their ear dermis (Dutta et al., 2012). Here, we were able to duplicate this finding in BALB/c mice and found it specific to the ear dermis, but not footpad or tail base. Translucency of ear due to its thinness may facilitate the efficiency of *in situ* illumination with white light, especially the short wavelength of the spectrum, i.e., ∼400 nm optimal for excitation of URO. Ear dermis was thus chosen as the site for PDV against challenge infection in the tail base. This choice is based not solely on the consideration of safety, but on that of efficacy. *Leishmania* subjected to double PS-sensitization alone without photo-inactivation *in vitro* was found to persist in comparable abundance in all three cutaneous sites (ear dermis, footpad, and tail base), but only those in the ear dermis were photo-inactivated beyond detection by *in situ* illumination (**Figure 4**). Thus, at this site there is an optimal amount of “vaccines” made available to APC via photo-inactivation of *Leishmania* therein and, more importantly, little or no immunosuppression by live parasites expected due to their virtual absence. By both accounts, PDV appears to create a microenvironment more favorably to elicit immunity in the ear dermis than tail base and foot pad. This immunity produced by PDV of the ear dermis is manifested against challenge infection by significantly delaying the onset of lesion development and by the marked reduction of parasite load seen in the challenging site at the end point of day 69 (**Figure 5**). This level of protection is significant, considering that most mouse lineages, including C3H, C57BL/6, BDA, and CBA all fail to heal or control *L. amazonensis* infection (Soong et al., 1997; Cortes et al., 2010), to which a susceptible phenotype of mixed Th1–Th2 response is often developed (Afonso and Scott, 1993; Mcmahon-Pratt and Alexander, 2004). BALB/c mice are especially susceptible to the infection by this species, presenting progressive development of non-healing necrotic lesion (Pereira and Alves, 2008). More complete protection by PDV is expected by optimizing the experimental conditions, e.g., reduction of the parasite dose for challenge infection, increasing the frequency of immunization more than once and/or adjustment of the time intervals between inoculation of doubly PS-sensitized *Leishmania* and *in situ* photo-inactivation. Optimization of this interval is expected to maximize photo-inactivation of doubly PS-sensitized *Leishmania*, thereby minimizing their migration from ear dermis to the draining lymph nodes. Minimization of this migration reduces the parasite population that escapes photo-inactivation by spot-illumination of the inoculation site, thereby reducing their immunosuppressive activities in the lymph nodes and facilitating immune clearance. The importance of immune clearance has been discussed previously and cannot be over-emphasized, considering its apparent necessity for post chemotherapeutic cure of leishmaniasis, since no drug is expected to reach every parasite in the patients, regardless of dosages used and treatment duration (Chang, 2014).

The current working hypothesis is that macrophages and DC take up *in vitro* doubly PS-sensitized *Leishmania* for subsequent cytolysis to release antigens therein after *in vivo* photo-inactivation, while the host APC remains viable and functional (Dutta et al., 2012). Therefore, antigen presentation and ensuing cellular-based immune response can be induced effectively, in keeping with the development of life long immunity known to occur after spontaneous or chemotherapeutic cure of human leishmaniasis. In summary, results presented demonstrate that PS-sensitization and photo-inactivation of *L. amazonensis* based on a combination of endogenous and exogenous strategies renders them non-viable, but immunologically competent against CL. The results presented remain to be a proof-of-principle, pending further investigation to reduce the complexity of PS-sensitization and photo-inactivation. Work is underway toward the simplification of these steps to facilitate the standardization and scale-up production of such photodynamically inactivated vaccines.

## Author Contributions

Conceived and designed the experiments: SV, CdO, and KC. Performed the experiments: SV, FC, and LR. Analyzed the data: SV, FC, BK, CdO, and KC. Contributed reagents/materials/analysis tools: DN, BK, and KC. Wrote the manuscript: SV, BK, KC, and CdO.

## Conflict of Interest Statement

The authors declare that the research was conducted in the absence of any commercial or financial relationships that could be construed as a potential conflict of interest.
