# Supplementary material for: Photodynamic Vaccination of BALB/c Mice for Prophylaxis of Cutaneous Leishmaniasis Caused by Leishmania amazonensis
Source: Front Microbiol. 2018 Feb 6;9:165. doi: 10.3389/fmicb.2018.00165 (PMC5808246; doi:10.3389/fmicb.2018.00165)
Supplement: Supplementary file 1 [file Presentation_1.PDF]

Photodynamic vaccination of BALB/c mice for prophylaxis of cutaneous leishmaniasis caused by *Leishmania amazonensis*

Sayonara M. Viana[1], Fabiana S. Celes[1], Laura Ramirez[1], Bala Kolli[2], Dennis Ng[3], Kwang Poo Chang[2,*], Camila I. de Oliveira[1,4*]

Supplemental Material

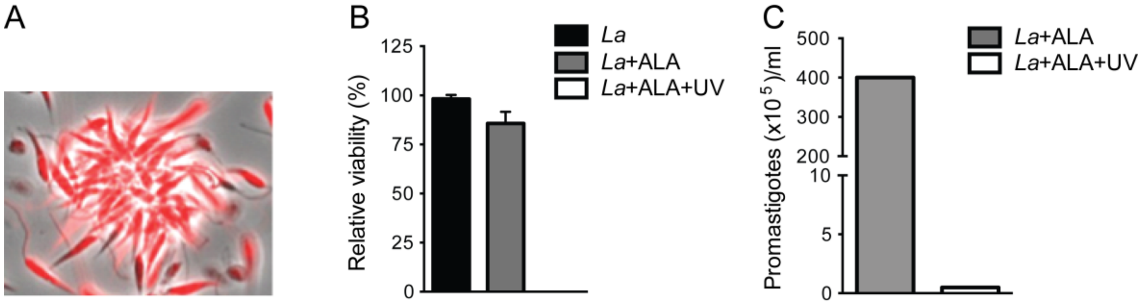

Supplemental Figure 1 – Viability of transgenic *L. amazonensis* induced with ALA for uroporphyrin accumulation followed by light exposure. *L. amazonensis* (**La**) genetically complemented to express ALAD and PBGD were incubated in the dark with ALA (1 mM) (+**ALA**) for 24 h and exposed to longwave UV light for 20 min (+**UV**). **[A]** A merged image captured first under phase contrast and then under porphyrin filter for fluorescence, showing cytosolic URO accumulation throughout the cells. **[B]** MTT reduction activities of untreated cells (**black bar**), those treated with ALA alone (**gray bar**) and in combination with UV light (**blank**). **[C]** Disparity between *La*+ALA and *La*+ALA+UV in cell density after incubation in culture medium for 7 days. Data are presented as mean ± S.D. from a representative experiment set performed in quadruplicate.

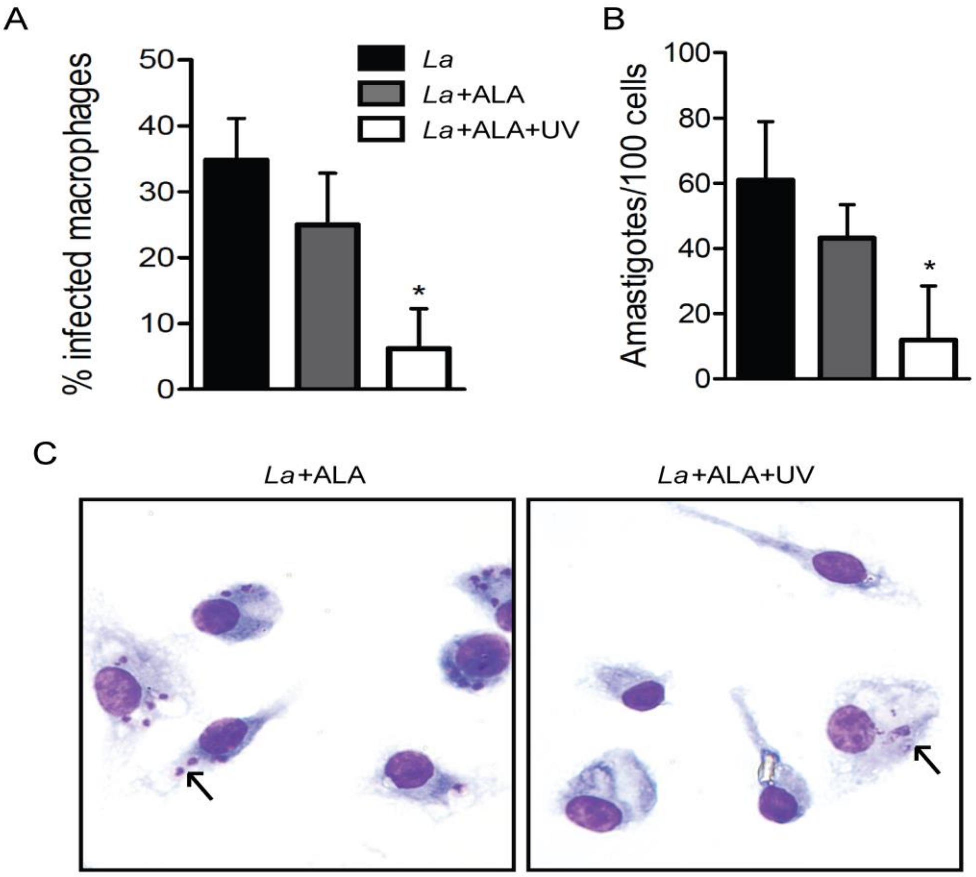

Supplemental Figure 2 – **Reduced uptake of uroporphyric and photo-inactivated *L. amazonensis* by primary macrophages.** Bone marrow-derived macrophages were infected at a host to parasite ratio of 1:10 for 4h with untreated *L. amazonensis* (**black bar**), those ALA-sensitized alone (**gray bar**) and in combination with exposure to UV light (**blank bar**). Cells were processed for microscopy to determine: **[A]** the percentage of infected macrophages; and **[B]** the number of *Leishmania* per 100 macrophages. **[C]** Representative photomicrographs of cultures shown in A and B. Data are presented as mean ± S.D from a representative experiment set performed in quadruplicate (Kruskal-Wallis test, *$p<0.05$). Arrow, Endocytosed *Leishmania*.
